# Fluoroalkyl Amino Reagents (FARs): A General Approach towards the Synthesis of Heterocyclic Compounds Bearing Emergent Fluorinated Substituents

**DOI:** 10.3390/molecules22060977

**Published:** 2017-06-12

**Authors:** Bruno Commare, Etienne Schmitt, Fallia Aribi, Armen Panossian, Jean-Pierre Vors, Sergiy Pazenok, Frédéric R. Leroux

**Affiliations:** 1 University of Strasbourg, CNRS, LCM UMR 7509, 67000 Strasbourg, France; bcommare@unistra.fr (B.C.); schmitt_etienne@msn.com (E.S.); fallia.aribi@gmail.com (F.A.); armen.panossian@unistra.fr (A.P.); 2 Bayer S.A.S., 14 Impasse Pierre Baizet, BP 99163, 69263 Lyon CEDEX 09, France; jean-pierre.vors@bayer.com; 3 Bayer AG, Alfred-Nobel-Strasse 50, 40789 Monheim, Germany; sergiy.pazenok@bayer.com

**Keywords:** fluorine, FAR, heterocycles, fluoroalkyl, difluoromethyl, emergent fluorinated substituents

## Abstract

Fluorinated heterocycles are important building blocks in pharmaceutical, agrochemical and material sciences. Therefore, organofluorine chemistry has witnessed high interest in the development of efficient methods for the introduction of emergent fluorinated substituents (EFS) onto heterocycles. In this context, fluoroalkyl amino reagents (FARs)—a class of chemicals that was slightly forgotten over the last decades—has emerged again recently and proved to be a powerful tool for the introduction of various fluorinated groups onto (hetero)aromatic derivatives.

## 1. Introduction

The incorporation of fluorine or fluorinated moieties into organic compounds plays a key role in life science-oriented research, as it can often result in profound changes to the physico-chemical and biological properties of the resulting compounds [1]. Therefore, organofluorine chemistry has become a new challenge in the context of small-molecule research in agro- [2,3,4,5,6,7,8] and medicinal chemistry [9,10,11,12,13]. Consequently, extensive and increasing attention has been devoted in the last decades to the development of new and more efficient methods for the introduction of fluorinated motifs. Classic methods for rapid assembly of fluoralkyl-substituted compounds rely almost exclusively on the commercial availability of fluorinated building blocks that are manufactured by Swarts-type reactions, a method for which no industrially viable substitute existed up to recently. Indeed, an alternative strategy emerged in the last decade in industrial scale applications, based on the use of fluoroalkyl amino reagents (FARs) as new tools to introduce fluoroalkyl moieties. This review will cover the preparation and the reactivity of FARs as well as their numerous applications.

## 2. Preparation and Properties of Fluoroalkyl Amino Reagents 

### 2.1. Preparation and Availability

Following the discovery of polytetrafluoroethylene (PTFE) by Plunkett in 1938, early examples of *N*,*N*-dialkyl α,α-difluoroalkylamines made from fluorinated alkenes were reported right after the Second World War. Indeed, the first reaction between nucleophiles and chlorotrifluoroethylene was reported for the first time in 1950 by Pruett et al. [14]. Then, Knunyants et al. reported in 1956 the addition of several nucleophiles, including secondary amines, on perfluoropropene [15]. In 1959, Yarovenko et al. described for the first time the preparation and application of 2-chloro-*N*,*N*-diethyl-1,1,2-trifluoroethan-1-amine (**1b**), later called the Yarovenko reagent, for deoxyfluorination of alcohols [16]. In 1960, England et al. reported a broad extension of the scope of a number of base-catalyzed additions to fluoro-olefins [17]. Although already synthesized by the Knunyants group, Ishikawa et al. described in 1979 the preparation of *N*,*N*-diethyl-1,1,2,3,3,3-hexafluoropropan-1-amine (**1c**) by condensation of perfluoropropene and diethylamine [18]. Based on previous work from the England group, Petrov et al. described completely the preparation of 1,1,2,2-tetrafluoro-*N*,*N*-dimethylethan-1-amine (**1a**, TFEDMA, sometimes called Petrov’s reagent) in 2001 [19]. Recently Walkowiak et al. reported the preparation of other FARs from 1,1,3,3,3-pentafluoropropene and various secondary amines to study the influence of alkyl chains of the secondary amine on the HF elimination process [20].

**Scheme 1:**
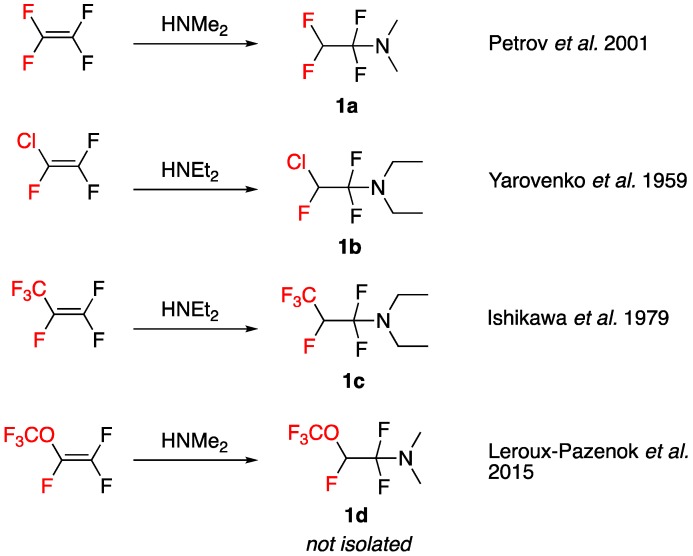
Preparation of fluoroalkyl amino reagents (FARs)—hydroamination of polyfluoroalkenes.

Nowadays, Petrov’s reagent (**1a**), Yarovenko’s reagent (**1b**) and Ishikawa’s reagent (**1c**) are commercially available from many suppliers, but their syntheses remain unchanged. FARs are still prepared by hydroamination of polyfluoroalkenes with secondary amines, which are both bulk chemicals produced on ton-scale in the fluoropolymer industry. This represents an advantage, as both ingredients for the preparation of FARs are rather cheap. TFEDMA (the Petrov reagent) can be purchased in a relatively high purity (>97% wt.) and the use of this yellow liquid is very convenient. Yarovenko’s reagent is a dark brown oil (available with 97% wt. purity), whereas the Ishikawa reagent is a pale brown oil with lower purity (ca. 90% wt.). Both are less stable than TFEDMA and degrade much more rapidly. Their purity must be measured prior to use by means of NMR analysis in strictly anhydrous, non-protic and non-nucleophilic deuterated solvents (e.g., CD_3_CN). One should indeed have always in mind that these FARs have to be handled under inert-gas atmospheres, as they are moisture sensitive, and their hydrolysis results in the release of hydrofluoric acid (HF). In 2015 a new FAR, (CF_3_OCFHCF_2_N(CH_3_)_2_) **1d** was developed by Leroux and Pazenok [21,22] for the introduction of CHFOCF_3_ as a challenging emergent fluoroalkyl substituent. It can be prepared in situ under its activated form (see next section) from commercially available gaseous trifluoromethyl trifluorovinyl ether. The new fluoroalkoxyfluoroalkyl group is highly electron withdrawing and has lower steric hindrance than CHFCF_3_ (in Ishikawa’s reagent) due to the oxygen spacer between the CF_3_ moiety and the reactive electrophilic center (Scheme 1).

### 2.2. Lewis Acid Activation of Fluoroalkyl Amino Reagents 

**Scheme 2:**
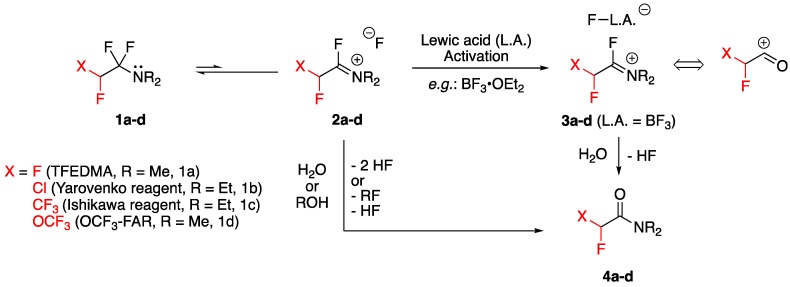
Lewis acid-mediated activation of FARs. TFEDMA, 1,1,2,2-tetrafluoro-*N*,*N*-dimethylethan-1-amine.

FARs show a unique reactivity due to the presence of highly electron-withdrawing fluorine atoms located closely to the tertiary amine. Indeed, the negative hyperconjugation resulting from an overlap of the filled non-bonding orbital of nitrogen with an empty anti-bonding orbital of the C–F bond weakens the latter to generate an equilibrium between the amines **1a**–**d** and the fluoroiminium forms **2a**–**d**, although intermediates **2a**–**d** could never be observed directly. This phenomenon is responsible for the specific reactivity of FARs. The difluoroalkylamine/fluoroiminium equilibrium can be fully shifted to the iminium form after activation by a Lewis acid, yielding iminium tetrafluoroborate salts **3a**–**d** in case of BF_3_·OEt_2_. These intermediates display a powerful electrophilic reactivity similarly to an acylium ion (Scheme 2). They also have some structural analogy with well-known iminium salts, such as the Vilsmeier reagent [23].

**Scheme 3:**
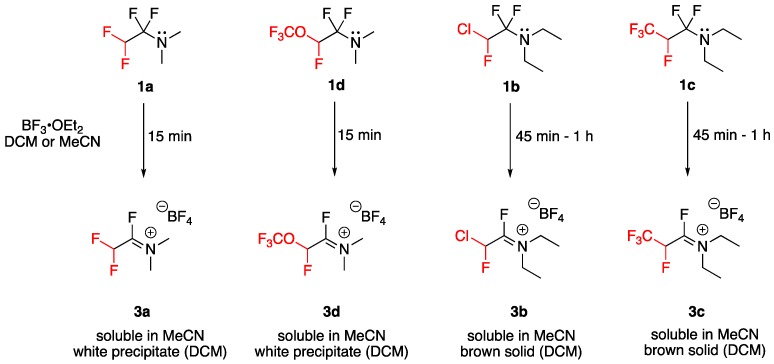
Activation of FARs with BF_3_·OEt_2_.

Both fluoroiminiums (fluoride **2a**–**d** or tetrafluoroborate **3a**–**d** salts) are highly moisture sensitive; they release hydrogen fluoride in contact with air to afford the corresponding fluorinated acetamides **4a**–**d**. The activation of FARs with Lewis acids is usually carried out in DCM or MeCN. The activated form is soluble in MeCN whereas it precipitates in DCM; evaporation of the latter solvent allows to isolate the fluoroiminium salt which is stable for a few hours under inert atmosphere (only for a few minutes under air). TFEDMA **1a** and “OCF_3_-FAR” **1d** can be used quite conveniently without this precipitation step; thus, they can be activated directly in MeCN over 15 min, as this solvent usually constitutes the medium for further reactions. However, due to their lower purity and slower activity, the Yarovenko **1b** and Ishikawa **1c** reagents are activated over 45 min to 1 h and are preferably used after precipitation from DCM (Scheme 3).

Concerning the choice of the Lewis acid, boron trifluoride diethyl etherate (BF_3_·OEt_2_) and aluminium (III) chloride are commonly used with a preference for the first one. Indeed, the activation of TFEDMA with AlCl_3_ in MeCN is slightly longer than with BF_3_·OEt_2_ (1 h instead of <15 min). The resulting counter-anion is also important in the reactivity of FARs. Tetrahedral BF_4_^−^ is less nucleophilic and basic than nitrates and halides and tetrafluoroborate salts are usually more soluble in organic solvents. Experimentally, FARs are usually rather simple to use on small-scale reactions even though small quantities of hydrogen fluoride are released. Simple glassware was conveniently used without excessive corrosion. Teflon flasks are however used when reactions are carried out on large scale (>10 g scale).

## 3. Fluoroalkyl Amino Reagents: Efficient Tools for Fluorination and for the Transfer of Fluoroalkyl Groups

### 3.1. General Reactivity Modes of FARs

**Scheme 4:**
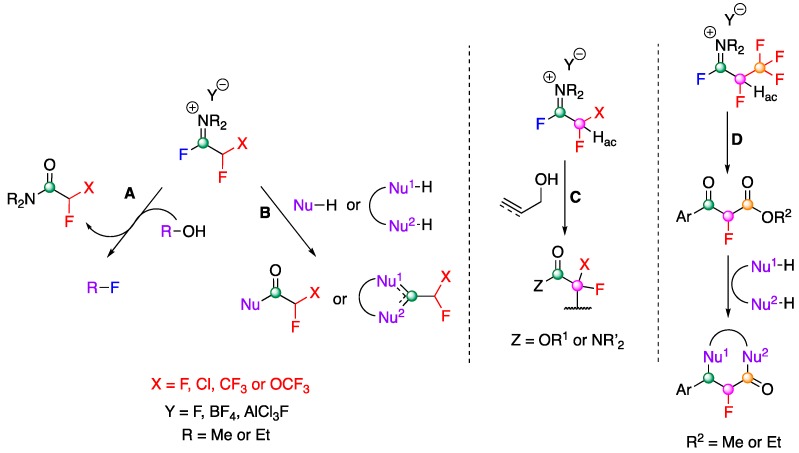
Overview of the diverse reactivity modes and applications of FARs.

The high electrophilicity of FARs, especially in their activated iminium form, and their ability to release hydrogen fluoride confer them a specific reactivity, which can be divided in four modes (Scheme 4) depending on the substrates and on the FAR used:

(A) No carbon of the FAR is incorporated in the desired product of the reaction. The FAR acts as an activator of hydroxyl groups, leading to their replacement by fluorine (with release of the hydrolysed FAR as a fluorinated acetamide) or another intramolecular nucleophile as in an example of Beckmann rearrangement. Aldehydes can also be deoxofluorinated. (Section 3.2).
(B) All carbons of the FAR are present in the desired product of the reaction but only one, the carbon of the iminium, undergoes transformations via one or two nucleophilic attack(s). This reactivity mode concerns the acylation of aromatic derivatives (Section 3.3.) and the synthesis of fluorinated heterocycles by ring-closing attacks of heteroatomic nucleophiles (Section 3.4).
(C) All carbons of the FAR are present in the desired product of the reaction and 2 carbons, the carbon of the iminium and the methine in α position, undergo transformations. This kind of reactivity is observed when nucleophiles are either allylic or propargylic alcohols (Section 3.5).
(D) All carbons of the FAR are present in the desired product of the reaction and all of them, namely the carbon of the iminium, the α-methine and the carbon in β position (CF_3_) undergo transformations. Accordingly, this reactivity is observed only with the Ishikawa reagent (Section 3.6).

### 3.2. Nucleophilic Fluorination of the Hydroxyl or Carbonyl Functions

**Scheme 5:**
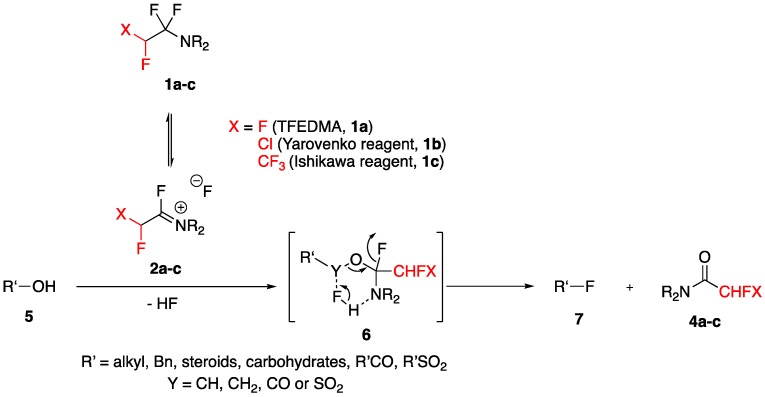
Dehydroxyfluorination— mechanism proposed by Petrov et al. [19].

Since the 1960s till today, the Petrov reagent (**1a**), the Yarovenko reagent (**1b**) and the Ishikawa reagent (**1c**) have been commonly used as selective fluorination agents of compounds containing a hydroxyl moiety, such as alcohols [18,19,24,25,26,27,28,29,30,31,32,33,34,35,36,37,38,39,40,41,42,43,44,45,46,47,48,49], including hydroxyproline [50,51,52,53] or carbohydrate derivatives [54], sulfonic [19] and carboxylic acids [55,56,57,58]. Interestingly, carbonyl compounds can also react with FARs to afford difluoromethylated compounds [59]. The mechanism consists in the formation of intermediate **6** as a result of the reaction between the hydroxyl function and the fluoroiminium followed by the decomposition of intermediate **6** to afford the fluorinated product **7** and the corresponding fluorinated acetamide **4a**–**c** (Scheme 5).

The Yarovenko [60] and the Ishikawa [61] reagents were also used to prepare amide compounds thanks to their capacity to provide efficiently acyl fluorides from carboxylic acids. They have also found applications as dehydrating agents to prepare acetylenic ketones from β-diketones [62]. TFEDMA can be reacted with 1,3-linear diketones (enolizable ketones) to provide β-difluoroketones [63].

**Scheme 6:**
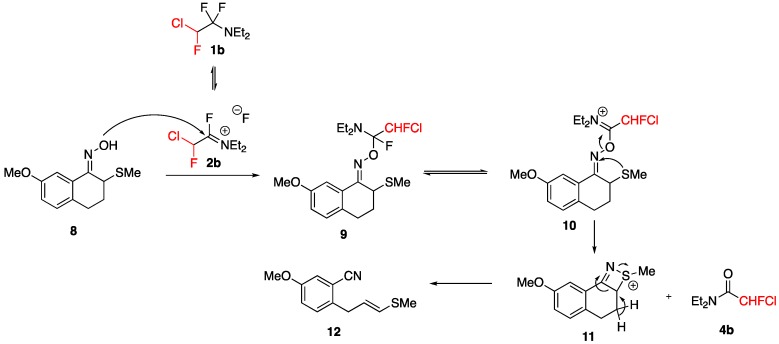
Beckmann rearrangement initiated by the Yarovenko reagent.

Finally, the Yarovenko reagent was also used to trigger the Beckmann rearrangement of α-methioxyketoxime (Scheme 6) [64,65]. Indeed, the hydroxylamine can react with the Yarovenko reagent to form intermediate **9**. Then, instead of undergoing an attack by the fluoride anion, as for usual reactions of alcohols with FARs, intermediate **10** engages in an intramolecular addition of the sulfur atom, releasing the fluorinated acetamide **4b** and leading to a thiazete **11** which finally fragments.

### 3.3. Acylation of Aromatics

**Scheme 7:**
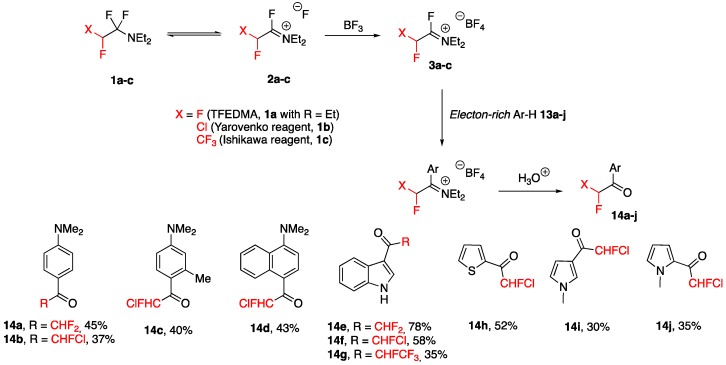
Acylation of electron-rich aromatics with FARs by Wakselman et al. [66].

The chemistry of FARs enriched in 1975, when Wakselman et al. used them for the fluoroacylation of electron-rich aromatics and more precisely, of dimethylaminobenzene, naphthalene, indole, thiophene and *N*-Me-pyrrole (compounds **13a**–**j**) using the activated forms of TFEDMA (**3a**), Yarovenko’s reagent (**3b**) and Ishikawa’s reagent (**3c**) in a Friedel-Crafts-type reaction. After hydrolysis of the resulting arylcarbiminium salt intermediate, acylated aromatics **14a**–**j** were isolated in moderate to good yields (Scheme 7) [66].

**Scheme 8:**
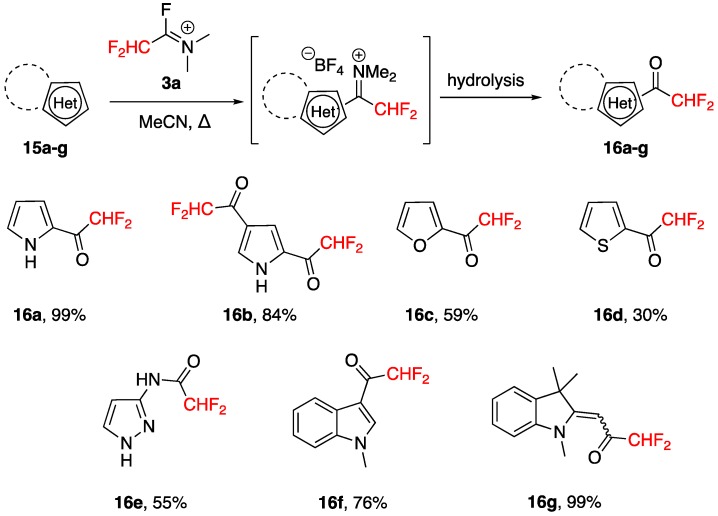
Difluoroacylation of electron-rich heterocycles with TFEDMA.

This method was recently extended to other heterocycles, such as pyrrole, furan, thiophene or *N*-methylindole (**15a**–**g**). For example, pyrrole was efficiently difluoroacylated and the introduction of a second difluoroacyl group was achieved to provide **16b** with high yield. Furan **16c** and thiophene **16d** gave lower yields, due to a high volatility and sensitivity towards hydrolysis or decomposition. 3-Aminopyrazole reacted via nucleophilic attack of its most nucleophilic position, namely the amino function, to afford the corresponding amide **16e**. *N*-methylindole and trimethylmethyleneindoline led to the corresponding derivatives **16f** and **16g**, respectively (Scheme 8) [67].

**Scheme 9:**
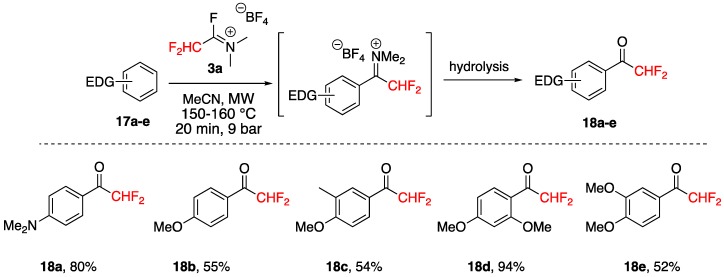
Difluoroacylation of electron-rich arenes under microwave heating. EDG, electron-donating group; MW, microwave.

Although thermal conditions usually used give good results, microwave assistance allows one to achieve much shorter reaction times and higher yields. Several difluoroacylated aniline and anisole derivatives **18a**–**e** could be isolated with moderate to excellent yields and with a regioselectivity governed by the substituents, as in usual S_E_Ar (electrophilic aromatic substitution) reactions (Scheme 9) [67].

**Scheme 10:**
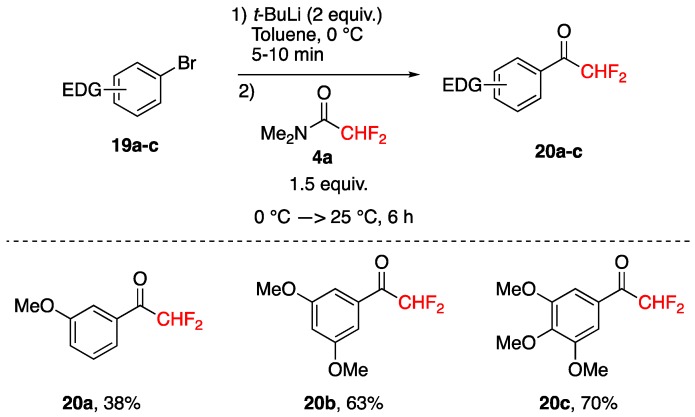
Difluoroacylation of electron-rich arenes with non-S_E_Ar (electrophilic aromatic substitution) regioselectivity by Br/Li exchange followed by trapping with a difluoroacetamide.

In order to access the other regioisomers and to broaden the substrate scope, halogen/metal exchanges can be employed to convert aryl halides into the desired difluoroacyl derivatives. Indeed, nucleophilic species formed in situ after the bromine/lithium exchange can be trapped with *N*,*N*-dimethyldifluoroacetamide (**4a**, obtained by hydrolysis of TFEDMA) to afford the difluoroacylated carbocycles **20a**–**c** (Scheme 10) [67].

A few examples of C-fluoroacylation of non-aromatic substrates were also reported. Whereas non-cyclic 1,3-diketones undergo deoxofluorination (Section 3.2), the cyclic analogues react with TFEDMA to yield the product of fluoroacylation of the active methylene [63]. Finally, we can notice that in presence of a tertiary amine (*N*,*N*-diisopropylethylamine (DIPEA) for example), some alkyl alcohols react with the Ishikawa reagent to afford the corresponding α-perfluoroesters, i.e., the products of O-acylation instead of the usual dehydroxyfluorination [68]. Similar results were obtained with aliphatic β-nitroalcohols [69] and α-halogenocyclohexanols [70], even in absence of additional base.

### 3.4. Synthesis of Fluoroalkylated Heterocycles

#### 3.4.1. Synthesis of Mono-Fluoroalkylated Benzo-Fused Heterocycles from 1,2-Diheteroatom-functionalized Arenes

**Scheme 11:**
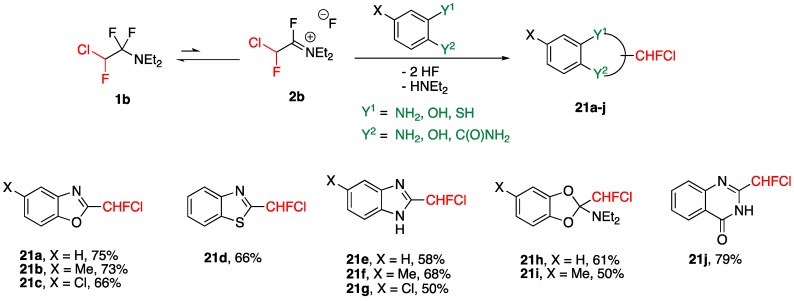
Synthesis of fluoroalkylated benzo-fused heterocycles without activation of FARs by Ishikawa et al. [71].

In 1979, the group of Ishikawa described the first FAR-based preparation of fluoroalkylated heterocycles, such as benzimidazoles, benzothiazoles and quinazolones from the Yarovenko reagent **1b**. New heteroarene compounds **21a**–**j** bearing a CHFCl group are produced with yields ranging from 50 to 75% (Scheme 11) [71].

These first results demonstrate the powerful potential of FARs to transfer fluoralkyl groups and access to various fluorinated (hetero)arenes which are ubiquitous in life science-oriented research.

#### 3.4.2. Synthesis of Mono-Fluoroalkylated Pyrazoles

**Figure 1:**
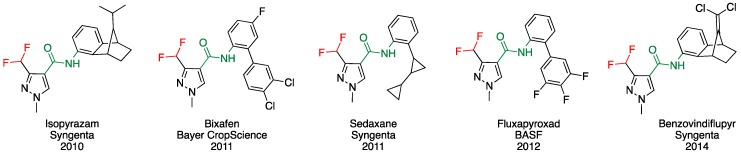
Launched succinate dehydrogenase inhibitors (SDHIs) based on fluorinated pyrazolecarboxamides.

Since the beginning of the 21st century, difluoromethylpyrazoles [72] have attracted considerable attention in crop science, since the 3-CHF_2_-pyrazolecarboxamide motif is actually found in new-generation top selling succinate dehydrogenase inhibitor (SDHI) fungicides (Figure 1) [72,73,74,75,76,77].

Whereas synthetics approaches towards pyrazoles bearing “classical” fluorinated substituents (F and CF_3_) have been widely studied and reviewed by Fustero et al. [78], the introduction of fluoroalkyl groups other than CF_3_ onto various N-based heterocycles is still the focus of intense research interest. In 2008, Pazenok et al. reported the utilization of TFEDMA for the preparation of ethyl 3-(difluoromethyl)-1-methyl-1*H*-pyrazole-4-carboxylate (DFMMP), the key intermediate of Bixafen^®^ (a modern SDHI fungicide) [79]. This first example of use of a FAR to access fluoroalkylpyrazoles prompted further investigation on FAR chemistry, as a means to develop new synthetic methods towards *N*-based heterocyles bearing emergent fluorinated substituents (EFS).

##### Towards the 3-CHF_2_-Pyrazolecarboxamide Motif

**Scheme 12:**
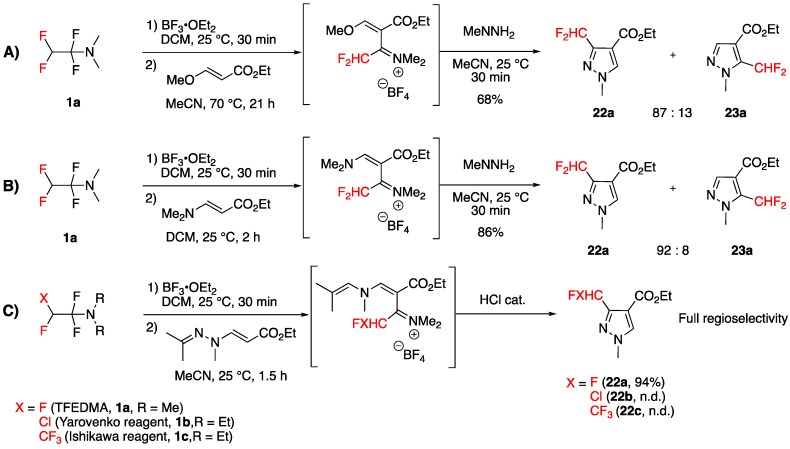
Use of FARs ((**A**) Reaction of TFEDMA with methoxy acrylate; (**B**) with dimethylamino acrylate; (**C**) with a methylhydrazone) and acrylates to prepare ethyl 3-(difluoromethyl)-1-methyl-1*H*-pyrazole-4-carboxylate (DFMMP) with full regioselectivity.

Several methods are described in the literature to prepare fluoroalkylpyrazoles. Most of them consist in the use of fluorinated precursors derived from difluoroacetic acid and subsequent cyclisation with hydrazines. All these methods were already reviewed in 2013 [80]. Another way consists in the construction of the fluoroalkyl group on the already formed pyrazole ring, by nucleophilic fluorination of chloroakyl or formyl groups or reductive dechlorination of chlorofluoroalkyl groups [81]. The first preparation of the desired DFMMP intermediate **22a** (Scheme 12) was patented in 1992 and was carried out starting from ethyl difluoroacetoacetate [82]. The product was obtained with good yield (74%), but the lack of regioselectivity and the difficult access to the starting material at this time (its availability is easier now) were major drawbacks of this first attempt. Several approaches have been described later to optimize the synthesis of DFMMP with full regioselectivity, high yield, low cost or non-toxic conditions which may be applied industrially. However, it was difficult to combine all these parameters.

To meet all required specifications, a new strategy was employed, based on the use of a specific FAR, namely TFEDMA (**1a**). The initial attempt involved the nucleophilic attack of ethyl 3-methoxyacrylate on activated TFEDMA to form the resulting iminium in situ, which was further cyclized by treatment with methyl hydrazine to afford the targeted DFMMP with 68% yield and a 87:13 ratio of isomers (Scheme 12A) [83]. This partial regioselectivity can be explained by the competition of two electrophilic centers during the attack by the hydrazine, resulting from the delocalization of the positive charge along the conjugated system. The ratio could be improved to 92:8 by replacing ethyl β-methoxyacrylate by ethyl β-dimethylaminoacrylate (Scheme 12B) [79]. Finally, full regioselectivity and high yield (94%) were obtained when the in situ formed fluoroiminium tetrafluoroborate salt was reacted with the protected hydrazine analogue of ethyl β-dimethylaminoacrylate (Scheme 12C) [84]. The preparations of CF_3_CHF- and CHFCl-functionalized analogues were successfully carried out using the same strategy (yields are not reported).

##### Synthesis of Various Substituted Mono (Fluoroalkyl)pyrazoles and Isoxazoles

**Scheme 13:**
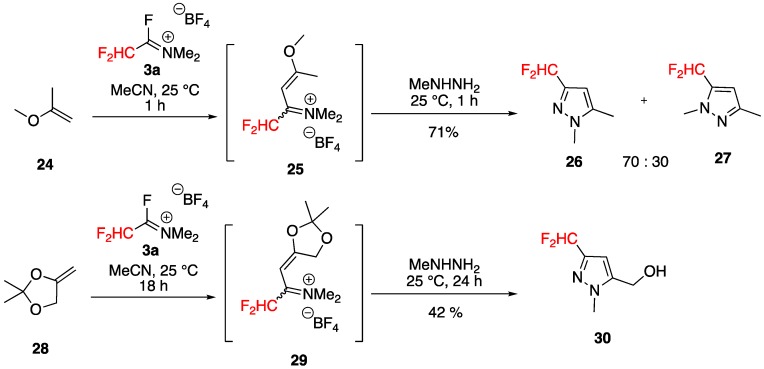
Reaction of activated TFEDMA **3a** with vinyl ethers.

As described above, activated FARs reacted well with amino- or alkoxyacrylates to form in situ highly reactive dielectrophilic species, precursors of mono(fluoroalkyl)pyrazoles. As a logical extension, the reactivity towards other nucleophiles was studied to prepare several substituted mono(fluoroalkyl)pyrazoles and -isoxazoles [67]. First, activated TFEDMA **3a** can react smoothly with vinyl ethers **24** and ketene acetals **28** to form iminium intermediates **25** and **29** and afford corresponding substituted mono(CHF_2_)-*N*Me-pyrazoles **26**, **27** and **30** after cyclization with methyl hydrazine (Scheme 13).

**Scheme 14:**
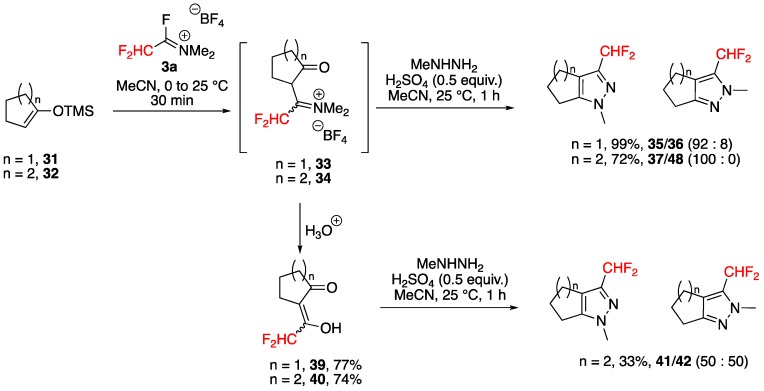
Reaction of **3a** with silyl enol ethers of cyclopentanone and cyclohexanone.

Second, investigations about the reactivity of **3a** with silyl enol ethers were conducted [67]. Commercial silyl enol ethers of cyclopentanone **31** and cyclohexanone **32** can react with fluoroiminium salt **3a** affording CHF_2_-iminium intermediates **33** and **34**, which can be either used directly in cyclization or hydrolyzed to isolate the corresponding β-(2,2-difluoro-1-hydroxy-ethylidene)cycloalkyl ketones **39** and **40**. When treated with methyl hydrazine, **40** gave a 1:1 mixture of regioisomers **41**/**42**, whereas iminium intermediates **33** and **34** led to the major isomers **35** and **37** with very good to complete regioselectivity (Scheme 14). This difference of regioselectivity can be explained by the higher electrophilicity of the iminium carbon in **33** and **34** with regard to the same carbon of enolic type in **39** and **40** and to the carbonyl group.

**Scheme 15:**
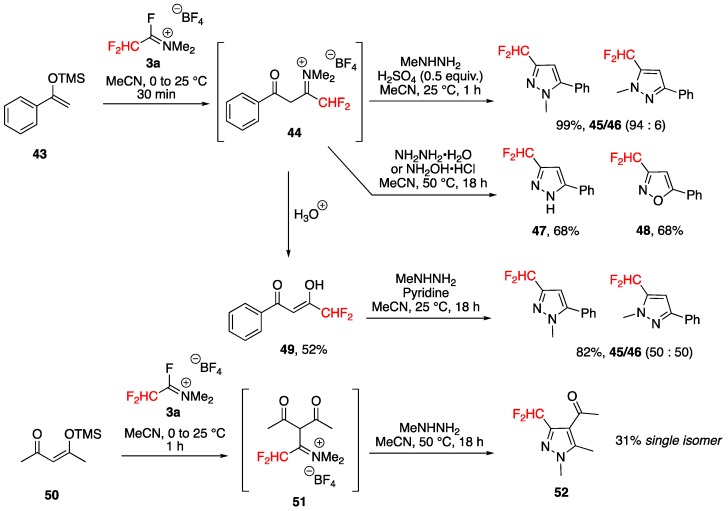
Reaction of **3a** with silyl enol ethers of acetophenone and acetylacetone.

These differences of regioselectivity were equally observed with the silyl enol ether of acetophenone **43** affording the major isomer **45** with very good selectivity (94:6) when avoiding hydrolysis of the iminium intermediate **44**. The latter was able to react also with hydrazine hydrate and hydroxylamine hydrochloride to provide NH-pyrazole **47** and isoxazole **48** respectively. The silyl enol ether of acetylacetone **50** afforded a single acetyl pyrazole isomer **52** (Scheme 15) [67].

**Scheme 16:**
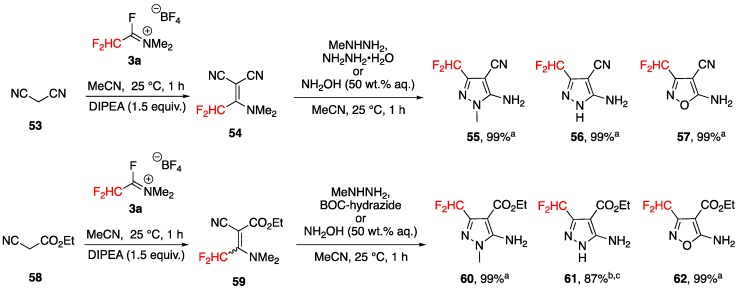
Preparation of difluoromethyl 5-aminopyrazoles- and isoxazoles. ^a 19^F-NMR yield using PhF as internal standard. ^b^ isolated yield. ^c^ 40 °C, 18 h. DIPEA, *N*,*N*-diisopropylethylamine.

Third, 3-difluoromethylpyrazoles and -isoxazoles bearing an amino group in position 5 can be obtained by reacting activated TFEDMA **3a** and CH-acidic nitrile derivatives, namely malononitrile **53** and ethyl cyanoacetate **58**, and following with a cyclization step with hydrazines or hydroxylamine (Scheme 16). The implementation of the first stage of the reaction proved delicate. The choice of the base and the isolation of the intermediate difluoro(dimethyamino)ethylidenes **54** and **59** appeared critical. However, the cyclization step was much easier and afforded efficiently 3-difluoromethyl-5-aminopyrazoles (**55**, **56**, **60** and **61**) and -isoxazoles (**57** and **62**) in presence of corresponding dinucleophiles (BOC-hydrazide (BOC, *tert*-butoxycarbonyle) was used instead of hydrazine hydrate in the case of compound **61** in order to improve the efficacy of the reaction) [67]. Last, monofluoroalkylpyrazoles could also be prepared by reaction of activated FARs and azines; this strategy will be described in Synthesis of 3,5-Bis(fluoroalkyl)-NH-pyrazoles from Azines Section.

#### 3.4.3. Synthesis of Bis-fluoroalkylated Pyrazoles

The huge diversity of targets in crop science and the success of DFMMP derivatives (Figure 1) motivated the search for analogues of this key motif bearing an additional fluoroalkyl group on the pyrazole ring.

##### Synthesis of 3,5-Bis(fluoroalkyl)pyrazoles from Fluoroacetoacetates

**Scheme 17:**
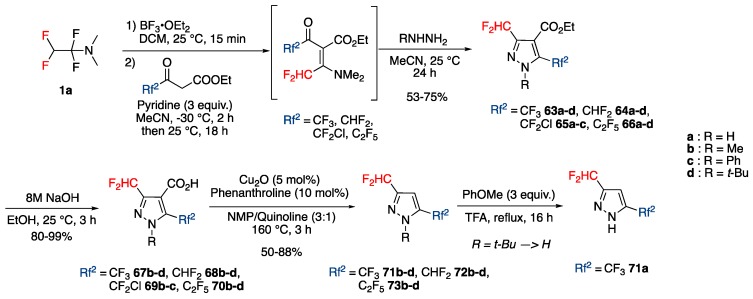
First preparation of 3,5-bis(fluoroalkyl)pyrazolecarboxylates and -carboxylic acids and their decarboxylation to afford 3,5-bis(fluoroalkyl)-NH-pyrazoles. TFA, trifluoroacetic acid; NMP, *N*-methyl-2-pyrrolidone.

Previous work already described the synthesis of pyrazoles bearing two fluorinated groups by reaction of bisperfluoroalkyl diketones with hydrazines, but the synthesis, isolation and purification of the starting fluorinated diketones is very complex [85,86,87,88,89,90]. To circumvent these issues, FARs proved a very valuable tool and allowed to develop a scalable and operationally convenient method. Indeed, they could act as a source of one fluoroalkyl group, while the other one was provided by available fluoroacetoacetates, leading after treatment with hydrazines to 3,5-bis(fluoroalkyl)-pyrazolecarboxylates **63**–**66** with excellent regioselectivity (>97:3) using a one-pot procedure (Scheme 17) [91]. This method could be applied on 100 g scale without any problems related to exothermicity or stirring [92]. In the case of N-substituted pyrazoles, esters **63**–**66** could be further functionalized by saponification, yielding carboxylic acids **67**–**70** as possible precursors for the synthesis of pyrazolecarboxamides towards SDHI ingredients (see Figure 1), and an additional decarboxylation step led to 3,5-bis(fluoroalkyl)pyrazoles **71**–**73** unsubstituted in position 4. On the other hand, the saponification conditions failed on NH-pyrazoles. Consequently, an alternative pathway was used to access to “naked” 3,5-bis(fluoroalkyl)-NH-pyrazole **71a** via cleavage of the *N*-*t*Bu moiety of *N*-*t*Bu-3,5-bis(fluoroalkyl)pyrazoles in harsh acidic conditions prior to decarboxylation (Scheme 17) [91,92].

**Scheme 18:**
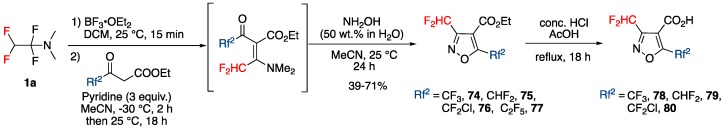
First preparation of 3,5-bis(fluoroalkyl)isoxazolecarboxylates and carboxylic acids.

The strategy was also used by Leroux and coworkers for the synthesis of 3,5-bis(fluoroalkyl)isoxazolecarboxylates **74**–**77** by replacing hydrazines with hydroxylamine. The corresponding carboxylic acids **78**–**80** were also prepared similarly by hydrolysis, although the latter was carried out in acidic medium (Scheme 18).

##### Synthesis of 3,5-Bis(fluoroalkyl)-NH-pyrazoles from Azines

**Scheme 19:**
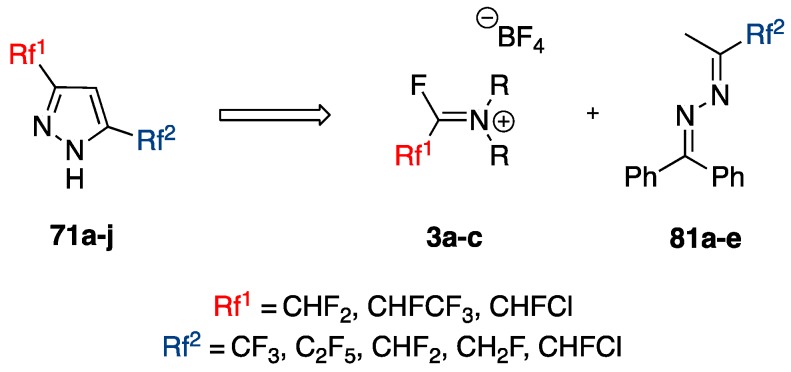
The fluorinated azine-based strategy to access 3,5-bis(fluoroalkyl)-NH-pyrazoles.

The method described above gave very good results in the access to 3,5-bis(fluoroalkyl)-pyrazolecarboxylates **63**–**66**. However, it suffered some limitations in the preparation of 3,5-bis(fluoroalkyl)-NH-pyrazoles **71a**—harsh acidic conditions were needed to deprotect the *N*-*t*Bu moiety. To circumvent this inconvenience and prepare efficiently unprecedented 3,5-bis(fluoro-alkyl)-NH-pyrazoles **71a**–**j**, another pathway was developed, based on the use of fluorinated azines **81a**–**e**. The latter are a synthetic equivalent of fluorinated propanyl-2-ylidenehydrazines, whose free NH_2_ is revealed upon in situ hydrolysis of the benzophenone-derived imine subunit. By reaction with activated FARs **3a**–**c** followed by addition of acid, a cyclization would occur to provide the desired NH-pyrazoles (Scheme 19) [93].

**Scheme 20:**
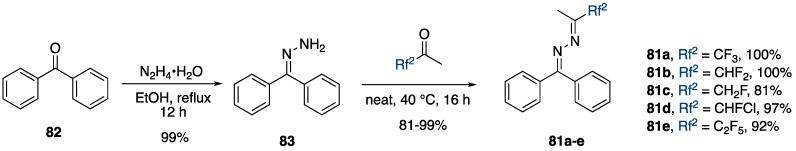
Preparation of fluoroacetone-derived azines from benzophenone hydrazine.

The preparation of fluororinated azines **81a**–**e** was straightforward. First benzophenone hydrazone **83** was prepared quantitatively by reaction of hydrazine hydrate with benzophenone **82**. Then fluoroacetones were condensed onto **83** to afford azines **81a**–**e** with excellent yields (Scheme 20) [93].

**Scheme 21:**
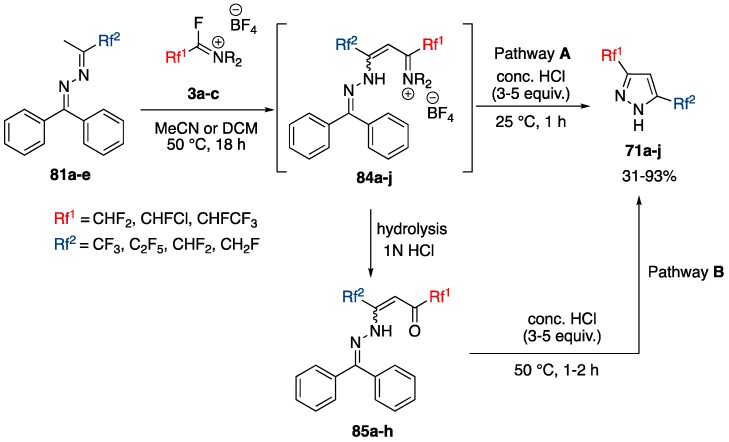
Synthesis of novel 3,5-bis(fluoroalkyl)-NH-pyrazoles. Pathway **A**: from in situ formed vinamidiniums; Pathway **B**: from isolated vinamides.

**Scheme 22:**
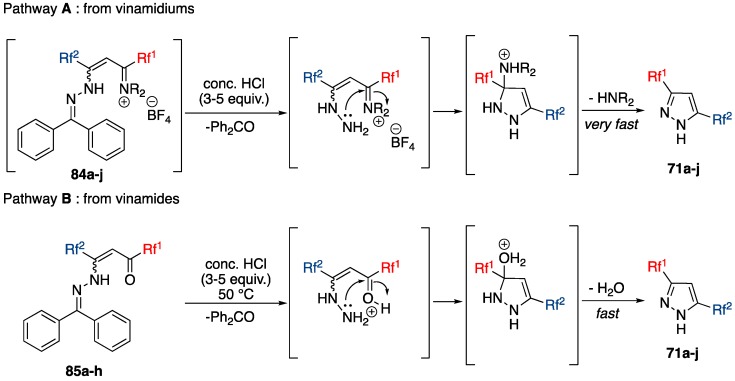
Supposed mechanism for the intramolecular cyclization from vinamidinums. (Pathway **A**) or vinamides (Pathway **B**).

Then, fluorinated azines **81a**–**e** were reacted with activated FARs **3a**–**c** (activation with BF_3_·OEt_2_) to form vinamidinium intermediates **84a**–**j**. On the one hand, the latter led, upon hydrolysis by dilute aqueous HCl (1 N), to β-(diphenylmethylenehydrazinyl)-bis(fluoroalkyl)-enones **85a**–**h**, which represent analogues of unsymmetrical fluorinated 1,3-diketones that are usually difficult to prepare. On the other hand, treatment of **84a**–**j** with concentrated HCl (12 N) hydrolyzed the benzophenone imine moiety and triggered the ring-closing attack of the resulting hydrazine onto the electrophilic β-fluoro iminium. This step provided the desired 3,5-bis(fluoroalkyl)-NH-pyrazoles **71a**–**j** with moderate to excellent yields (Scheme 21, pathway A). Interestingly, several of these pyrazoles could also be prepared from vinamides **85a**–**h**, by treating them with concentrated HCl, to compare the reactivity of vinamides versus vinamidiniums. Whereas the cylization proceeded smoothly at room temperature from vinamidiniums **84a**–**j**, heating the vinamides **85a**–**h** at 50 °C for 1–2 h was necessary to afford the cyclized products with lower yields (Scheme 21, pathway B). This difference in reactivity can be ascribed to the faster release of the secondary amine rather than that of water during the final aromatization step (Scheme 22) [93].

**Scheme 23:**
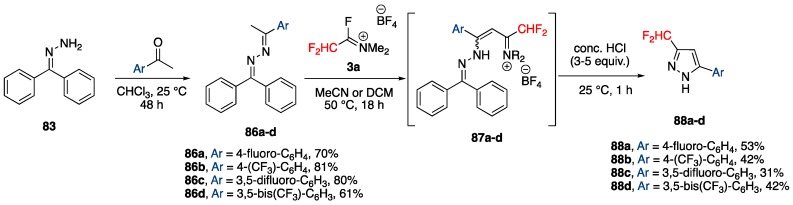
Synthesis of unprecedented 3-(CHF_2_)-5-(fluoroaryl)-NH-pyrazoles.

This strategy was also used to prepare 3-(CHF_2_)-5-(fluoroaryl)-NH-pyrazoles **88a**–**d** from fluorinated acetophenones **86a**–**d** with moderate yields (Scheme 23) [93].

This use of fluorinated azines represents the first efficient pathway to prepare unprecedented 3,5-bis(fluoroalkyl)-NH-pyrazoles. However, application of this method in industrial processes appears difficult, due to the tediousness of the complete removal of benzophenone released in the reaction. Moreover, the method was limited to the preparation of 3,5-bis(fluoroalkyl)-NH-pyrazoles. Several attempts of N-methylation of these compounds were achieved and proved that the regioselective N-functionalization is really difficult and mostly influenced by thermodynamic factors (unpublished results). Consequently, a new facile and efficient method was then reported to prepare series of 3,5-bis(fluoroalkyl)pyrazoles bearing not only a hydrogen or a methyl substituent, but also a large diversity of groups in position 1, while maintaining the control of regioselectivity [21]. This method will be described in the following sections.

##### Synthesis of 3,5-Bis(fluoroalkyl)-NH-pyrazoles from Ketimines

**Scheme 24:**
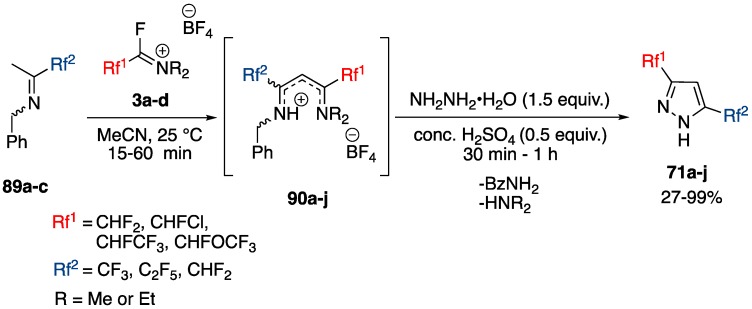
Synthesis of 3,5-bis(fluoroalkyl)-NH-pyrazoles from fluorinated ketimines and hydrazine hydrate.

This new strategy was based on the addition of *N*-benzyl fluoroacetimines **89a**–**c** on activated FARs **3a**–**d.** The reaction could be carried out under mild conditions (25 °C in MeCN for up to 1 h) to produce vinamidium intermediates **90a**–**j**. These species can be directly reacted with hydrazine hydrate to afford 3,5-bis(fluoroalkyl)-NH-pyrazoles **71a**–**j** under similarly mild conditions with moderate to excellent yields (Scheme 24). Interestingly, better results were attained with this ketimine-based method than with the azine-based route when starting from TFEDMA **1a**. The trifluoromethoxy-subsituted FAR **1d**, transferring a CHFOCF_3_ group, was also used and afforded new pyrazole scaffolds with very good yields (81–85%). On the other hand, the Yarovenko and Ishikawa reagents proved overall less efficient (except when starting from the CHF_2_-ketimine) due once again to the lower reactivity of *N*,*N*-diethyl iminiums with regard to their dimethyl congeners, and to the lower purity of the starting commercial FARs **1b**–**c** [21].

##### Synthesis of 3,5-Bis(fluoroalkyl)-NMe-pyrazoles from Ketimines

**Scheme 25:**
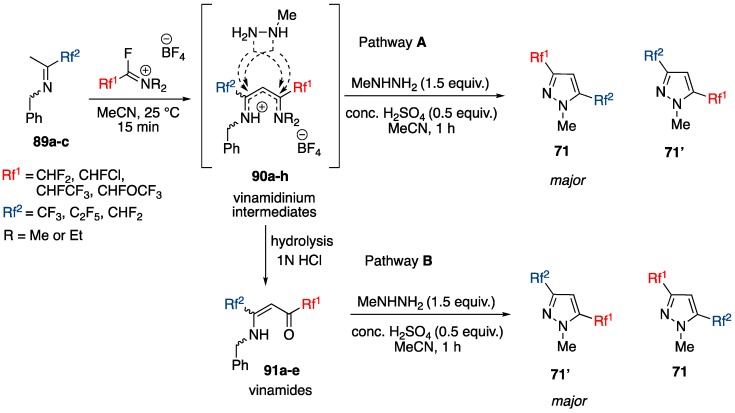
Synthesis of 3,5-bis(fluoroaryl)-*N*Me-pyrazoles. Pathway **A**: from in situ formed vinamidiniums; Pathway **B**: from isolated vinamides.

Unlike the synthesis of NH-pyrazoles from hydrazine hydrate, the access to NMe-pyrazoles implies an additional regioselectivity issue, due to the non-symmetrical nature of methyl hydrazine whose first nucleophilic attack can proceed via the NH_2_ or the NHMe groups (Scheme 25). The control of regioselectivity is critical since regioisomers **71** and **71′** are usually difficult to separate.

When vinamidinium intermediates **90a**–**j** were treated with methyl hydrazine under acidic conditions (Scheme 25, pathway A), the best results were again observed with TFEDMA **1a** and the “OCF_3_-FAR” **1d**, which led mainly to regioisomer **71** (**71**/**71′** ratio = 71:29 to 100:0). For example, full regioselectivity in favour of isomer **71** was observed when **3d** was opposed to CF_3_- and C_2_F_5_-ketimines. Conversely, the activated Yarovenko and Ishikawa reagents **3b**–**c** gave poorer results in terms of both reactivity and regioselectivity. Indeed, no reaction occurred with electron-poor and bulkier ketimines (Rf^2^ = CF_3_ and C_2_F_5_); and while it proceeded with the CHF_2_-ketimine, a lower selectivity was observed, sometimes surprisingly in favour of isomer **71′** [21].

To account for the regioselectivity, one can assume that in the case of TFEDMA and its -OCF_3_ analogue, which both lead to *N*,*N*-dimethyliminiums, the major isomer is formed due to two reasons. The first attack is believed to be more favorably affected by the NH_2_ moiety of methyl hydrazine, instead of the NHMe one, in order to avoid the steric clash between the methyl group and fluorinated substituents Rf^1^ or Rf^2^. Second, this first attack is driven by the release of the more volatile dimethylamine instead of benzylamine. On the other hand, for the two other FARs, the lower reactivity of more congested *N*,*N*-diethyliminium salts **3b**–**c** renders their attack by the NH_2_ group more difficult and affords mixed regioselectivities.

Vinamidiums **90a**–**j** can also be hydrolysed to afford vinamides **91a**–**e**, which can react afterwards with methyl hydrazine as 1,3-dielectrophiles (Scheme 25, pathway B). In this case, a reversed regioselectivity is observed with regard to the reaction of vinamidiniums. For example, treating unsymmetrical vinamide 4-(benzylamino)-1,1,5,5-tetrafluoropent-3-en-2-one (Rf^1^ = Rf^2^ = CHF_2_) with methyl hydrazine led to isomer **71′** as major product, presumably after initial addition of the NH_2_ moiety of methyl hydrazine onto the iminium tautomer which is more electrophilic than the carbonyl function of vinamides **91** [21].

##### Synthesis of 3,5-Bis(fluoroalkyl)-N-substituted-pyrazoles from Ketimines

**Scheme 26:**
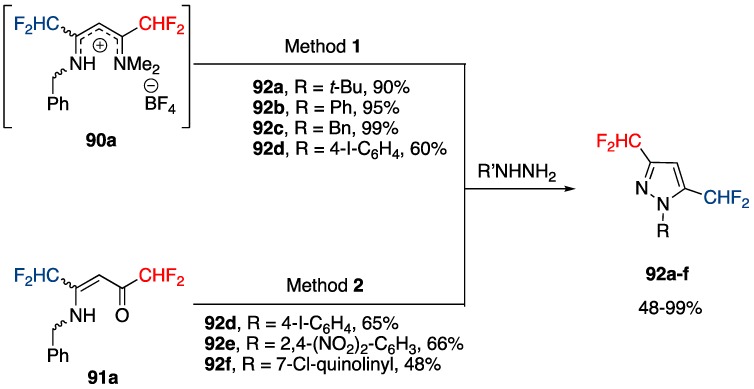
Synthesis of various N-substituted pyrazoles from vinamidiniums and vinamides. **Method 1**: hydrazine/conc. H_2_SO_4_, or hydrazine·HCl/NEt_3_, MeCN, 25–50 °C, 1 h.; **Method 2**: hydrazine, conc. H_2_SO_4_, toluene/MeCN, 120–140 °C, MW, 0.5–2 h.

After the development of efficient methods to prepare 3,5-bis(fluoroalkyl)-NH- and *N*Me-pyrazoles regioselectively, the synthesis of analogous pyrazoles bearing a wide diversity of substituents in position 1 was tackled, from commercially available substituted hydrazines. To avoid problems of regioselectivity, symmetrical bis(CHF_2_)pyrazoles were first prepared, by means of either hydrazine hydrochloride salts in presence of NEt_3_ (helping to solubilize salts and the aromatization), or free hydrazines in presence of sulfuric acid. Vinamidinium intermediate **90a** provided efficiently 1-alkyl- and 1-arylpyrazoles **92a**–**d** with very good yields (90–99%). For some hydrazines, especially the more hindered or more electron-deficient ones, microwave assistance was needed to afford the desired aryl pyrazoles **90d**–**f** with moderate yields (48–66%). Some limitations were observed, such as the non-compatibility of the reaction conditions with acid-labile groups on the final pyrazoles (BOC, tosyl, *t*Bu and benzoyl under certain conditions) or a sluggish mixture in the case of **92f**, but various *N*-substituted pyrazoles could still be obtained (Scheme 26) [21].

**Scheme 27:**
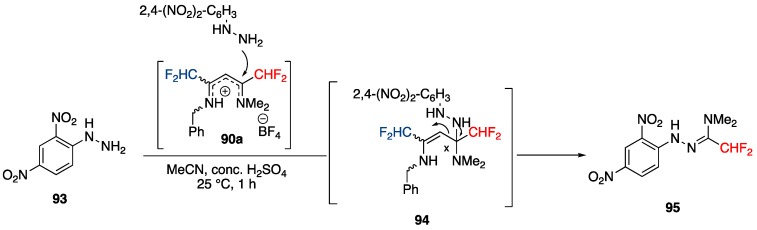
Observed side-reaction product with 2,4-dinitrophenylhydrazine.

Interestingly, when 2,4-dinitrophenylhydrazine **93** was reacted with vinamidinium **90a**, hydrazonamide **95** was formed in 83% yield, thus supporting the scenario where the first nucleophilic attack is effected by the NH_2_ end of the hydrazine onto the *N*,*N*-dimethyl iminium moiety of the vinamidinium (Scheme 27).

**Scheme 28:**
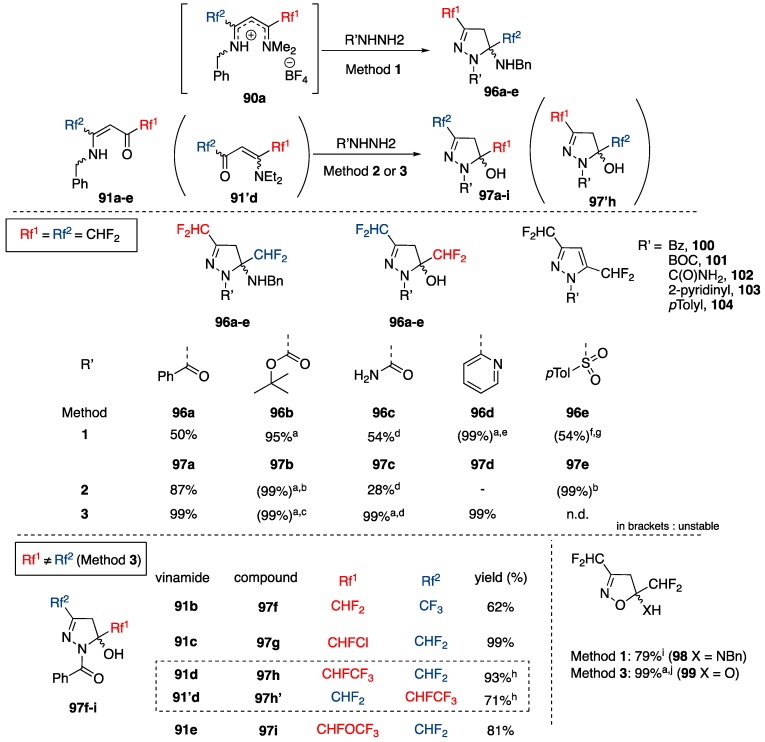
Regioselective preparation of 5-*N*-benzylamino- and 5-hydroxypyrazolines and isoxazolines. **Method 1**: hydrazine, conc. H_2_SO_4_, MeCN, 25–50 °C, 1 h. **Method 2**: hydrazine, toluene/MeCN, 120–140 °C, MW, 0.5–2 h. **Method 3**: hydrazine, HFIP (hexafluoropropan-2-ol), 100–140 °C, 0.5–5 h. ^a 19^F NMR yield with PhF as internal standard. ^b^ R group cleaved between 120 °C and 150 °C. bis(CHF_2_)-NH-pyrazole **71** formed. ^c^ R group cleaved between 80 °C and 120 °C. bis(CHF_2_)-NH-pyrazole **71** formed. ^d^ prepared from a mixture of semicarbazide hydrochloride and NEt_3_, with no acid added. ^e^ Pyrazole **103** was isolated directly. ^f^ No conc. H_2_SO_4_ used. ^g^ *N*-(*p*Tolyl)-pyrazole (**104**) was separated by chromatography from pyrazoline **96e** (29% isolated). ^h^ Pyrazolines **97h** and **97’h** were prepared from a 65/35 mixture of vinamides **91d** and **91’d** and separated by chromatography. ^i^ Hydroxylamine (50 wt. % aq.) used instead of hydrazine. ^j^ Hydroxylamine·HCl used instead hydrazine.

On the other hand, when hydrazines bearing a H-bonding N-substituent (benzoyl, BOC, carbamyl, 2-pyridinyl, tosyl), were used, the dehydration/deamination step (aromatization step) did not proceed and the corresponding hydroxy- or *N*-benzylaminopyrazolines were obtained (Scheme 28) [21]. As reported by several research groups, fluoroalkyl pyrazoles can be prepared from hydrazines and fluorinated 1,3-diketones or analogues, but the intermediate fluorinated 5-hydroxypyrazolines are often not dehydrated readily under the reaction conditions [94,95,96]. Since vinamidiniums **90** or vinamides **91** can be regarded as mono- or bis-iminium analogues of bis(fluoroalkyl)-1,3-diketones, it is not surprising that their reaction with hydrazines bearing a H-bonding N-substituent leads to non-aromatized products. Indeed, the latter substituent binds to the proton of the hydroxy or benzylamino group, thus increasing electron-density at O and N respectively, and therefore decreasing the acidity of the β-proton whose abstraction would lead to aromatization.

Several pyrazolines were thus isolated and demonstrated an excellent stability (Scheme 28) [21]. Interestingly, these experiments demonstrate the opposite reactivity of vinamidinium and vinamide intermediates. Indeed, 5-(*N*-benzylamino)pyrazolines **96a**–**e** were selectively prepared from bis(CHF_2_)-substituted vinamidinium **90a** (Method **1**) whereas 5-hydroxy-pyrazolines **97a**–**e** were obtained from the corresponding vinamide **91a** (Method **2**). These results seem again to indicate that the first nucleophilic attack is carried out by the less hindered NH_2_ moiety of hydrazines onto the *N*,*N*-dimethyl iminium part of vinamidinium **90a**, while, in vinamide **91a**, this attack takes place on the *N*-benzyl iminium instead.

Using the fluorinated polar protic solvent hexafluoropropan-2-ol (HFIP) involved a critical improvement in the reaction of vinamides (Method **3**). This non-nucleophilic and highly H-bonding solvent proved highly appealing in the preparation of 5-hydroxypyrazolines **97a**–**e** since it provided excellent yields in absence of strong Brønsted acid. This method was also used with non-symmetrical vinamides **91b**–**e** and for every Rf^1^/Rf^2^ couple, the reactivity of the *N*-benzyl iminium moiety formed in situ was always higher than that of the fluoroalkyl ketone function towards attack by the NH_2_ end of the hydrazine. Four different unsymmetrical 5-hydroxy-pyrazolines **97f**–**i** were selectively formed with yield ranging from 62 to 99%. Using a mixture of vinamides **91d/91′d** (65:35) provided respectively a mixture of 5-hydroxy-pyrazolines **97h/97′h** (68:32) further separated by chromatography with almost complete conservation of the initial ratio (Scheme 28) [21].

The synthesis of the corresponding isoxazolines was achieved similarly using aqueous hydroxylamine or hydroxylamine hydrochloride instead of hydrazine. 5-(*N*-benzylamino)isoxazoline **98** and 5-hydroxypyrazoline **99** were isolated in very good yields (Scheme 28) [21]. This demonstrates that the more nucleophilic nitrogen attacks the more electrophilic iminium group in both starting vinamidinium salt **90a** (*N*,*N*-dimethyl iminium) and vinamide **91a** (*N*-benzyl iminium). The stabilization of the non-aromatized isoxazoline is permitted by either 1,4-*H*-bonding interactions or intermolecular H-bonding interactions.

**Scheme 29:**
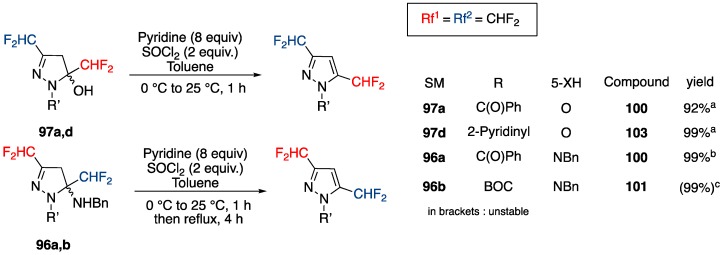
Dehydration of several pyrazolines in basic conditions; ^a^ Yield of isolated product. ^b 19^F NMR yield with PhF as internal standard. ^c^ bis(CHF_2_)-NH-pyrazole **73** formed after BOC (*tert*-butoxycarbonyle) cleavage.

Then, a selection of bis(fluoroalkyl)pyrazolines was successfully rearomatized under basic conditions (excess of pyridine) using thionyl chloride. *N*-benzoyl-5-hydroxypyrazoline **96** and *N*-2-pyridinyl-5-hydroxypyrazoline **97** were readily and quantitatively dehydrated at room temperature to yield the corresponding pyrazoles **100** and **103**. Conversely, reflux heating was required for the aromatization of *N*-benzoyl-5-(*N*-benzylamino)pyrazoline to provide pyrazole **100** and similarly for the *N*-(BOC)-analogue, which afforded quantitatively the bis(CHF_2_)-NH-pyrazole **71** due to the thermal instability of the BOC group (Scheme 29) [21].

To complete the investigation, a variety of functional groups (halogen, nitro, amine, aldehyde, carboxylic acid, boronate) was introduced into the 4-position of the model substrate, 3,5-bis(CHF_2_)-NH-pyrazole **71**, to improve the applicability of 3,5-bis(fluoroalkyl)pyrazoles [21].

#### 3.4.4. Synthesis of 2,4-Bis(fluoroalkyl)-substituted Quinoline Derivatives

**Scheme 30:**
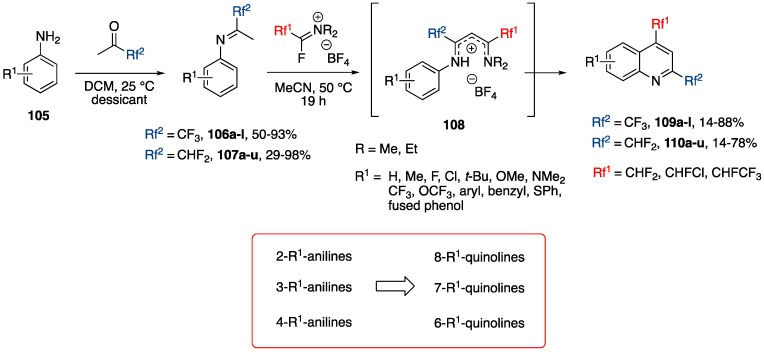
Preparation of 2,4-bis(fluoroalkyl)quinolines from aryl fluoroketimines.

The previous section covered the reaction of FARs with fluorinated *N*-benzylketimines to prepare 3,5-bis(fluoroalkyl)pyrazoles. When *N*-aryl fluoroketimines are used instead, the reaction outcome drastically changes. In this case, the vinamidinium intermediate readily cyclizes without addition of a hydrazine or of hydroxylamine as cyclization partner. The highly electrophilic distal fluorinated iminium indeed undergoes attack by the aryl substituent of the remote nitrogen, in a Friedel-Crafts-type reaction, to finally afford 2,4-bis(fluoroalkyl)quinolines after rearomatization. The synthesis of quinoline derivatives bearing two fluorinated groups in both positions 2 and 4 is scarcely described; only syntheses of bis(trifluoromethylated)quinolines were reported [97,98,99,100]. The use of FARs allowed to prepare in one step, from two series of variously substituted aryl fluoroketimines **106a**–**l** and **107a**–**u**, a large diversity of 2,4-bis(fluoroalkyl)quinolines **109a**–**l** and **110u** bearing different fluorinated groups on the pyrido moiety and various substituents on the benzo ring under mild conditions. Interestingly, complete regioselectivity was always observed, obviously with *N*-(4-substituted-phenyl)imines, but also with the 2- and 3- substituted analogues. The reaction yields were dependent on the nature of the substituents (R^1^), of the starting aniline of the Rf^1^ and Rf^2^ groups and the R substituents of the FAR nitrogen atom. Indeed, the critical intermediate **108**, where the nucleophilic and electrophilic termini required for cyclization are part of the same molecule and heavily conjugated, is strongly affected by the electronic and steric effects of all substituents decorating the *N*-aryl vinamidinium backbone (Scheme 30) [101].

### 3.5. Reaction with Allylic and Propargylic Alcohols

In previous Section 3.2, Section 3.3 and Section 3.4 we have described the uses of FARs to perform the dehydroxy-fluorination of alcohols, with no carbon of the FAR present in the final product, and reactions where all carbons of the FAR are present in the product but only one, the carbon of the iminium, undergoes transformation.

**Scheme 31:**
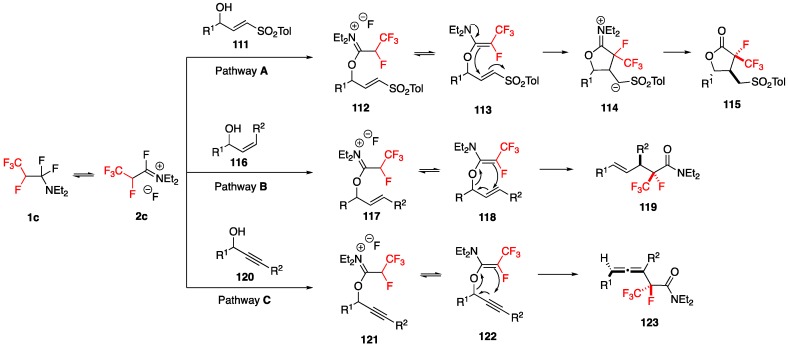
Reaction between the Ishikawa reagent and allylic or propargylic alcohols.

When allylic or propargylic alcohols are reacted with FARs, another, distinct outcome is revealed, with two carbons of the FAR being transformed and incorporated in the reaction product. Indeed, the reaction between the Ishikawa reagent **1c** and the hydroxyl function of allylic **111** and **116** or propargylic **120** alcohols affords iminium intermediates **112**, **117** and **121**. Due to the acidic proton in *α* position of the imidate carbon, the latter undergoes tautomery leading to the enamine form which can then react intramolecularly as a nucleophile to form different fluoralkylated molecules. Thus, α-fluoro-α-trifluoromethyl-γ-lactones **115** can be formed stereospecifically from Ishikawa’s reagent and racemic or enantioenriched γ-hydroxy-α,β-unsaturated sulfones **111** (Scheme 31, pathway A) [102]. The diastereoselective formation of 2-fluoro-2-trifluoromethyl-4-alkenamides **119** was also reported from **1c** and (*Z*)-allylic alcohols **116** via a Claisen rearrangement (Scheme 31, pathway B) [103]. The same technique was reproduced from propargyl alcohols **120** to afford the related allenes **123** with good yields (Scheme 31, pathway C) [104]. These reactions were then applied to the diastereoselective and enantioselective synthesis of α-trifluoromethylated α-amino acid derivatives from γ-hydroxy-α-fluoro-α-trifluoromethyl carboxamides [105]. In the end, although this reactivity mode of FARs has only been reported for the Ishikawa reagent, one can assume that other FARs can be compatible.

### 3.6. Transformation of the Three Carbons of the Ishikawa Reagent

**Scheme 32:**
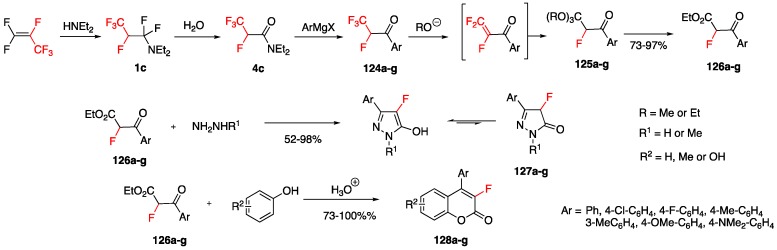
Synthesis of fluorinated heterocycles from the hydrolyzed Ishikawa reagent [106].

Finally, another application of FARs makes a constructive use of all carbons of the FAR, which are al transformed and incorporated in the reaction product. The Ishikawa reagent, like other FARs, can be easily hydrolysed to form the corresponding acetamide **4c**, which can then be treated with a polar organometallic species (ArMgX) to afford acylated products, as detailed in Section 3.3. The α position of this ketone is relatively acidic and can be deprotonated by an alkoxide, to form in situ the corresponding difluoromethylene upon elimination of fluoride. The transient β,β-difluoroenone then reacts quickly with excess alkoxide to afford α-fluoro-β-ketoesters **126a**–**g**. It is important to note that this step is possible only when starting from the Ishikawa reagent, which is the only FAR among **1a**–**d** to be derived from a 3-carbon alkene. The resulting α-fluoro-β-ketoesters **126a**–**g** possess two electrophilic sites and can react with dinucleophiles to provide fluorinated heterocycles. For example, monofluorinated pyrazoles and coumarins can be prepared by reaction with hydrazine and phenols respectively (Scheme 32) [106].

## 4. Conclusions

While fluoroalkyl amino reagents were discovered more than a half century ago, their utilization was really diversified in 1975 when Wakselman et al. published their first applications as fluoroacylating agents for aromatics. The chemistry of FARs underwent a second impulse at the beginning of the 21st century when the need for fluorinated heterocycle-based crop protection ingredients by agrochemical companies focused on difluoromethylpyrazoles. Indeed, 3-CHF_2_-pyrazolecarboxamide derivatives showed high activity as SDHI fungicides and several analogues were marketed by agro companies. In order to enhance the diversity and activities of these active ingredients, novel structures were sought and their preparation was studied. The development of new methods to introduce diverse emergent fluorinated substituents on heterocycles was necessary and FARs showed very interesting applications. Numerous fluorinated N-based 5- and 6-membered heterocycles bearing “classical” or new fluorinated substituents, particularly CF_3_, C_2_F_5_, CHF_2_, CHFCl, CHFCF_3_ or CHFOCF_3_ were successfully prepared using fast, efficient, robust and scalable methods.
